# Insights into immunogenicity and therapeutic strategies to mitigate the immune response in infantile-onset Pompe disease: a comprehensive systematic literature review

**DOI:** 10.3389/fimmu.2025.1690312

**Published:** 2026-01-08

**Authors:** Priya S. Kishnani, Johanna M. P. Van Den Hout, Andreas Hahn, David Kronn, Yin-Hsiu Chien, Mei Han, Jennifer Heuterman, Susan Sparks, Colin Glen, Nadia Daba

**Affiliations:** 1 Department of Pediatrics, Duke University Medical Center, Durham, NC, United States; 2 Centre for Lysosomal and Metabolic Diseases, Erasmus University Medical Center, Rotterdam, Netherlands; 3 Department of Child Neurology and Center of Rare Diseases Giessen, Justus-Liebig University, Giessen, Germany; 4 Departments of Pathology and Pediatrics, New York Medical College, Valhalla, NY, United States; 5 Department of Medical Genetics and Pediatrics, National Taiwan University Hospital, Taipei, Taiwan; 6 Sanofi, Cambridge, MA, United States; 7 Sanofi, Amsterdam, Netherlands; 8 Global Diagnostic and Scientific Affairs Department, Lucid Group, London, United Kingdom

**Keywords:** anti-drug antibodies, cross-reactive immunological material, enzyme replacement therapy, high sustained antibodies, infantile-onset pompe disease, pompe disease

## Abstract

**Introduction:**

Pompe disease, a rare autosomal recessive metabolic myopathy, is primarily treated with enzyme replacement therapy (ERT); however, ERT response depends on several factors, including ERT initiation age, dose, and cross-reactive immunological material (CRIM) status, especially in infantile-onset Pompe disease (IOPD).

**Aim:**

This systematic literature review (SLR) focused on three research questions (1): how CRIM status is determined in patients with IOPD in clinical practice, and how CRIM-negative status impacts outcomes (2); how health professionals use CRIM status to inform their decisions on immune tolerance induction (ITI) regimens; and (3) which regimens are used in real-world clinical practice.

**Methods:**

The SLR was conducted using Embase and PubMed databases covering the literature from January 1, 2003, to August 4, 2022. The search terms used were “Pompe or IOPD” and “cross-reactive immunological material or CRIM.” Data extraction was performed using pre-designed tables in Microsoft Excel. Among those identified, 54, 51, and 69 studies provided meaningful data for the respective research questions. The key theme was the importance of early diagnosis/treatment. Recently, there has been a major shift from direct CRIM testing using western blotting and mutation analysis to CRIM status prediction based on genetic variant analysis. The ITI regimen was mostly prescribed for CRIM-negative patients and some CRIM-positive cases in a prophylactic/naïve setting at ERT initiation to prevent the development of high antibodies and for IOPD patients irrespective of CRIM status in the ERT-experienced setting due to the presence of high and sustained anti-drug antibody levels. The frequently reported ITI regimen includes a short rituximab and methotrexate course in an ERT naïve setting, with/without intravenous immunoglobulin. CRIM-negative patients receiving ITI with ERT have better clinical outcomes than those not receiving the ITI regimen. Presently, the ITI regimen used in CRIM-positive patients is variable and based on physician preference, family history, or specific variants.

**Conclusion:**

The study concluded that CRIM status determination is important in patients with IOPD and impacts management approaches. ITI use has been predominantly reported in CRIM-negative patients to improve the clinical outcomes, with other important factors being early initiation of ERT and treatment above label dose of alglucosidase alpha and many are doing upto 40 mg/kg/2 weeks.

## 1 Introduction

Pompe disease is a rare autosomal recessive metabolic myopathy caused by a deficiency in the lysosomal glycogen-hydrolyzing enzyme, acid-α-glucosidase (GAA) (1). A recent study using data from newborn screening (NBS) between 2010 and 2022 reported the birth prevalence of Pompe disease as one per 18,711 births, i.e., 5.3 per 100,000 births across Asia, Europe, the United States (US), and South America (2). Based on the age of onset, Pompe disease is broadly classified into two subtypes: infantile-onset Pompe disease (IOPD), with the most severe being classic infantile Pompe disease, and late-onset Pompe disease (LOPD). Classic infantile Pompe disease is a rapidly progressive form that presents with severe cardiomyopathy and early death, whereas LOPD can manifest as early as the first year of life and is characterized by slower progression and the absence of hypertrophic cardiomyopathy (HCM) in the first year of life (3, 4).

Globally, IOPD occurs in approximately one per 150,000 births (5). IOPD is primarily characterized by a clinical signs, including hypotonia, progressive muscle weakness, and HCM in first year of life. It has low GAA activity in blood and <1% in the skin or muscle (1, 6). The clinical onset in infants with classic infantile-onset Pompe disease where HCM is noted prior to age 12 months (5, 7). These symptoms often progress rapidly, leading to respiratory and cardiac failure, which is generally fatal within the first year of life (6, 8).

Enzyme replacement therapy (ERT) is presently the standard lifesaving treatment for patients with Pompe disease (9, 10). Early initiation of ERT can improve cardiac, respiratory, and motor functions, thereby improving overall and ventilator-free survival in patients with IOPD (5). Alglucosidase alfa (ALG) was the first approved treatment for IOPD and LOPD in 2006 (11); avalglucosidase alfa (AVA), a next-generation ERT, was approved for LOPD patients greater than 12 months of age in the US in 2021 (12) and later for LOPD and IOPD in the European Union (EU) (13), Japan (14, 15), and Australia (16). Cipaglucosidase alfa plus miglustat is another next-generation ERT approved for LOPD in 2023 and is currently under clinical evaluation for IOPD and also in clinical trials as per the expanded access programme (NCT04327973) (17).

ERT improves the prognosis of patients by providing the missing enzyme and ameliorating the symptoms of Pompe disease (18, 19). However, the degree of response can vary based on several factors, including patient age at the time of treatment initiation, severity of symptoms at diagnosis, and dose used (cumulative, 20–40 mg/kg/2 weeks up to 40 mg/kg/week) (20, 21). In addition, the response of the body to protein-based therapeutics, such as ERT, may vary. In certain cases, the immune system may respond with the production of immunoglobulin G (IgG) anti-drug antibodies (ADAs) (22). These ADAs can potentially affect the safety and efficacy of protein-based therapies, which is a cause of concern (23). Its efficacy can change owing to altered pharmacokinetics and/or interference with activity. Patients with Pompe disease can present with very high anti-recombinant human acid alpha-glucosidase (rhGAA) IgG antibody titers (24), which can lead to a decline in the effect of therapy and infusion-associated reactions (25).

The cross-reactive immunological material (CRIM) status of patients with IOPD is an important aspect in predicting treatment response to ERT (26, 27). Patients with residual amounts of nonfunctional GAA protein on western blotting are classified as CRIM-positive, whereas patients with no detectable GAA protein are classified as CRIM-negative (27). There is a lack of immune tolerance to the administered rhGAA in CRIM-negative patients, whereas most CRIM-positive patients retaining some enzyme (nonfunctional/reduced in function) can recognize the rhGAA as self, whereas some behave like CRIM-negative patients. Thus, CRIM-negative patients and a subset of CRIM-positive patients (approximately 32%) have a poor response to ERT, mainly attributable to the development of high and sustained levels of ADAs. These responses can neutralize enzyme uptake into cells or the catalytic activity of the enzyme, thereby significantly reducing the effectiveness of treatment (27, 28). The neutralizing effect seems determined by the antibody: enzyme molecular stoichiometry (29). Following ERT initiation, CRIM-negative patients tended to seroconvert earlier than CRIM-positive patients (4 weeks, versus 12.7 weeks) (27, 28). High ADAs can develop even if ERT is initiated early, as noted in babies identified by NBS (30).

Given these complexities, immune tolerance induction (ITI) strategies have been developed to mitigate the immune response by preventing the development of ADAs and reducing their levels in response to ERT, thereby maintaining the effectiveness of treatment (30–33). Thus, based on patients’ experiences with ERT, different regimens to mitigate ITI are used (a naïve setting or an established/entrenched setting) (32, 34). In an ERT-naïve setting, prophylactic ITI can be administered using rituximab, low-dose methotrexate, and intravenous immunoglobulin (IVIg) for a short duration (35, 36). It is important to note that there is no delay in ERT initiation as prophylactic ITI regimen is done concurrently with ERT (30, 37). In ERT-experienced patients with an entrenched immune response, longer ITI regimens, including rituximab, methotrexate, IVIg, and plasma-cell targeting agents, such as bortezomib, have been used (30, 34, 38). Other therapeutic approaches for an entrenched setting include cyclophosphamide, methylprednisolone, or rapamycin (39–41).

The safety and efficacy of the ITI use in CRIM-negative patients have been reported globally (35, 36, 42). However, there is a need to increase understanding of how CRIM status influences ITI decisions and the real-world use of ITI regimens. The current article presents findings from a systematic literature review (SLR) that explores the following three research questions to understand the relationship between CRIM status, ITI regimens, and clinical outcomes in patients with IOPD.

Research question 1: How is CRIM status determined in clinical practice, and how does a CRIM-negative status with ERT alone impact the outcome in patients with IOPD?

Research question 2: How do healthcare professionals use CRIM status to inform their decisions on ITI regimens in patients with IOPD?

Research question 3: Which ITI regimens have been used in real-world clinical practice in patients with IOPD?

## 2 Methods

### 2.1 SLR

The SLR was conducted using the Embase and PubMed databases covering the literature from January 1, 2003, to August 4, 2022, and was in alignment with the Preferred Reporting Items for Systematic Reviews and Meta-Analyses (PRISMA) guidelines. The search terms used were “Pompe or IOPD” and “cross-reactive immunological material or CRIM.” This broad search strategy aimed to encompass all relevant literature pertaining to the three research questions, minimizing the risk of missing significant articles. The titles and abstracts of the articles identified through the searches were independently reviewed by two analysts. All identified articles were checked for relevance to the predefined inclusion criteria for each research question mentioned in **Table 1**. In cases of disagreement, the analysts conferred to reach a consensus. If unresolved, a third senior analyst was available for decision-making. Duplicate articles were identified and removed. The full texts of the articles that met the inclusion criteria were reviewed to confirm eligibility. Data extraction was conducted using pre-designed tables in Microsoft Excel. Non-English language papers were translated using Google Translate, and accuracy checks were conducted by a medical writer who was fluent in the original language. Statistical analyses were not performed.

**Table 1:** Screening criteria for research questions: CRIM status determination, ITI regimen decision-making, and clinical outcomes in patients with IOPD.

Study content	Research question 1^a^	Research question 2^b^	Research question 3^c^
Patients	IOPD (including subgroup analysis), any age, any sex, any country		
Interventions	Not applicable	Measurement, prediction, or use of CRIM status	ITI, any regimen, or none (including specific drugs such as bortezomib, IVIg, methotrexate, sirolimus, mycophenolate, and rituximab)
Comparators	Not applicable (reflects changes over time or across regions/countries)		Not applicable (reflects differences or similarities among regimens and with no ITI)
Outcomes	Methods of determining CRIM status (testing, prediction, or both)^d^	Report of use or non-use of ITI^e^ and/or regimens	Relationship of ITI^e^ use or regimen to CRIM status and various clinical outcomes^f^
Study design	Clinical studies	Clinical studies, including guidelines and algorithms	Clinical studies
Report	Articles published from January 1, 2003, to August 4, 2022^g^, in any language		
Exclusion criteria	Non-IOPD cases (e.g., LOPD only, unspecified Pompe disease) and pre-clinical studies		

a Research question 1: In patients with IOPD, how is the CRIM status determined in clinical practice?

b Research question 2: In patients with IOPD, how do healthcare professionals use CRIM status to inform their decisions on ITI regimens?

c Research question 3: In patients with IOPD, which ITI regimens have been used in real-world clinical practice, and how is their use (or lack thereof) associated with CRIM status and clinical outcomes?

d Full-text versions checked for CRIM status.

e No mention of ITI in a full journal article was considered to indicate no use of ITI; this was a change to the original protocol and was implemented by re-screening and reviewing previously excluded articles. In cases of not mentioned in the congress abstract, the report was not considered relevant to this research question.

f Mortality, respiratory function, mobility, muscle strength, motor function, cardiac function, biomarkers, antibody titers, and health-related quality of life.

g The outcomes of ERT clinical trials have been published primarily from 2003 onwards. This study was initiated in the third quarter of 2022; hence, the study period was from 2003 to 2022.

CRIM, cross-reactive immunological material; IOPD, infantile-onset Pompe disease; ITI, immune tolerance induction; IVIg, intravenous immunoglobulin; LOPD, late-onset Pompe disease.

### 2.2 Expert discussion

Two 2-hour advisory board meetings were organized with six experts from the US (*n =* 2), the Netherlands (*n* = 2), Germany (*n* = 1), and Taiwan (*n =* 1) in July and November 2023; results from the SLR report were presented and discussed in detail. The advisory board meetings were recorded and transcribed; the SLR report was updated based on these discussions.

## 3 Results

**Figure 1:**
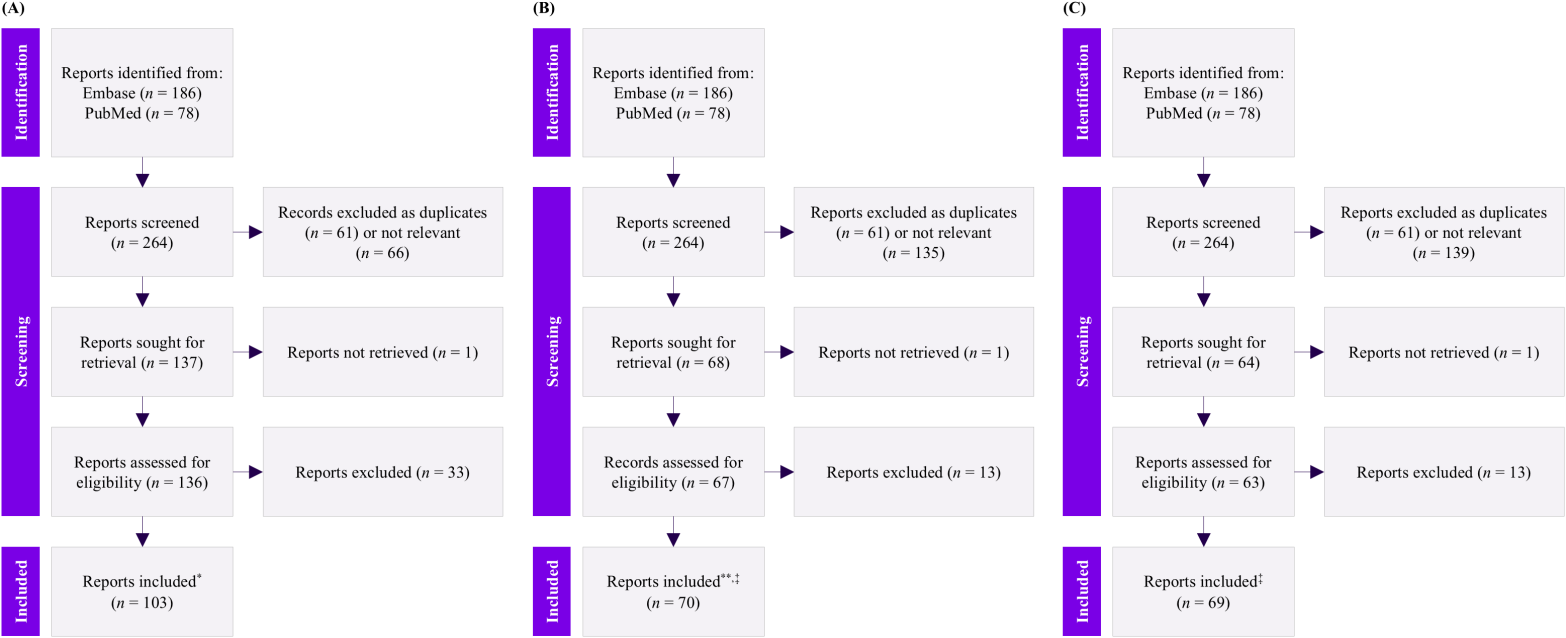
PRISMA flow diagram for research question 1 **(A)**, research question 2 **(B)**, and research question 3 **(C)**. *54 provided meaningful data out of the included 103 articles; **51 provided sufficient information for data extraction out of the included 70 articles. This determination was based on the relevance of the study’s methodological details; some of the reports were initially not included because they did not mention clinical decision-making methods, which were later found to be relevant. These full journal articles ^†^(*n* = 16) and ^‡^(*n =* 19) were subsequently included. *n*, number of studies; PRISMA, Preferred Reporting Items for Systematic Reviews and Meta-Analyses.

Systematic searches of the Embase and PubMed databases yielded 186 and 78 articles, respectively. After removing duplicates, screening, and full-text review, 103 reports were deemed potentially relevant for research question 1; 54 were further considered to provide meaningful data for analysis (**Figure 1A**). For research question 2, from a total of 70 articles included, 51 were deemed to provide meaningful analytical data (**Figure 1B**). For research question 3, all 69 articles included were considered to provide meaningful data for analysis (**Figure 1C**).

### 3.1 General findings

**Figure 2:**
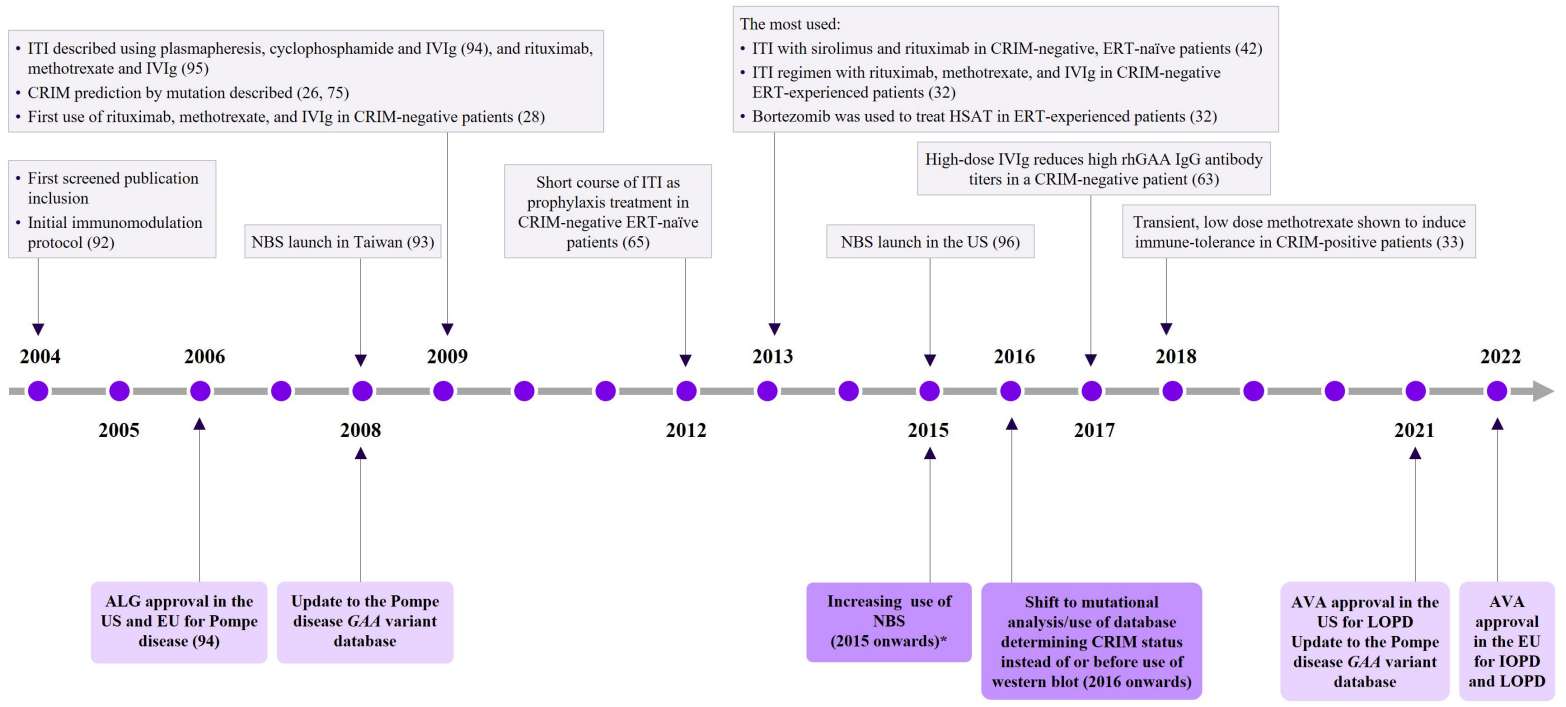
Summary of timeline pertaining to Pompe disease for the SLR (26, 31, 32, 41, 42, 50–56) *Pompe disease was added to the Recommended Uniform Screening Panel (RUSP) for the USA in 2015.ALG, alglucosidase alfa; AVA, avalglucosidase alfa; CRIM, cross-reactive immunological material; ERT, enzyme replacement therapy; EU, European Union; *GAA*, acid α-galactosidase; HSAT, high sustained antibody titers; ITI, immune tolerance induction; IOPD, infantile-onset Pompe disease; LOPD, late-onset Pompe disease; IVIg, intravenous immunoglobulin; NBS, newborn screening; rhGAA, recombinant human acid alpha-glucosidase; SLR, systematic literature review; US, United States.

**Figure 2** depicts the evolution of knowledge regarding the diagnosis and treatment of patients with IOPD, including the key initial ITI therapies. Over time, more ERTs and ITI regimens have become available to treat CRIM-negative and -positive patients with IOPD. The articles varied in terms of detail; however, the commonly reported details included patient characteristics such as low GAA enzyme activity, hypertrophic cardiomyopathy, muscle weakness, hypotonia, elevated serum creatinine, respiratory distress, and developmental delay. Some case studies have reported diagnoses either prenatally or shortly after birth, frequently triggered by cardiac symptoms (57–60). Furthermore, a number of GAA variants, including previously unclassified variants for CRIM status (c.2744A>C [p.Gln915Pro]) (61), (delT525/delT525) (18), and (c.1935C>A [p.D645E]) (46) have been reported. Overall, for research question 1, 63 reports described CRIM-negative patients with IOPD, and 64 reports described CRIM-positive patients with IOPD. Across all three research questions, most studies were conducted in the US or Europe (research question 1: 35.9% or 26.2%; research question 2: 45.7% or 25.7%; research question 3: 44.3% or 27.5%), respectively.

### 3.2 Evolution of CRIM status determination

Previous publications mentioned CRIM testing using methods such as western blot analysis of skin fibroblasts and pathogenic variants as the preferred method for CRIM status determination; however, the test results may take a long time, with a wait time of up to several weeks (26, 62). To overcome the long wait time with western blot analysis of skin fibroblasts, techniques like blood-based CRIM assay and mutation analysis are used to determine the CRIM status of the patients; these techniques yield quicker results within 48–72 hours (63). Recently, a trend towards increased usage of CRIM prediction by utilizing *GAA* variant analysis has been observed, as this leads to early treatment initiation and superior patient outcomes. However, the results of both techniques (blood-based CRIM assay and variant analysis) need to be interpreted with thorough knowledge and expertise. In a study conducted by Bali et al. (62) on 33 patients with IOPD, the CRIM status in peripheral blood mononuclear cells of 27 patients were in alignment with the CRIM status predicted by *GAA* pathogenic variants, but discordant or indeterminant in 6 patients, highlighting the need to validate the accuracy of the result obtained with blood-based assay alone and the importance of having the pathogenic variants to help further confirm CRIM status (62).

### 3.3 Determination of CRIM status in clinical practice (research question 1)

#### 3.3.1 Testing to determine CRIM status

Among the 103 reviewed articles, 49 were excluded because of insufficient information (**Figure 1A**). The key studies for research question 1 are summarized in **Table 2**.

**Table 2:** Summary of key studies for research question 1.

Author (year)	Number of patients	Dose	CRIM status determination	Outcomes
Bali et al. (2012) (26)	243	NA	Western blot analysis in skin fibroblasts, along with mutational analysis	Power of *GAA* gene mutations in predicting CRIM status
Banugaria et al. (2013) (31)	7	20 mg/kg eow to 20 mg/kg/weekly	GAA mutation analysis, confirmed by western blot analysis	Efficacy and safety of ITI with ERT therapy
Corti et al. (2014) (43)	Case study	20 mg/kg every 7–14 days	Western blot analysis	Immune response to AAV vectors
Markic et al. (2014) (44)	Case report	Dose varied over time from 20 mg/kg, thereafter 40 mg/kg, and finally 20 mg/kg eow	Western blot analysis	Clinical decline in CRIM-positive
Broomfield et al. (2015) (45)	33	ERT dose varied in patients from 20 mg/kg eow to initial 3 months 20 mg/kg/week, followed by 20 mg/kg eow and 40 mg/kg/week, ERT dosing 3: 40 mg/kg/week	CRIM assays	ERT treatment experience in a cohort of CRIM-negative patients
Chien et al. (2015) (46)	10	Variable	Western blot analysis	NBS and detection of disease on time
Berrier et al. (2015) (47)	20	Cumulative dose of 20–40 mg/kg e2w	Western blot analysis	Immune responses in CRIM-negative patients receiving ERT monotherapy
Sheets et al. (2015) (48)	2	NA	–	Discordant clinical responses. Suppression of HSATs with ITI
Gupta et al. (2020) (49)	77	20 mg/kg eow	*GAA* variant information and/or through Western blot analysis	Overall survival and survival probability in patients who received and did not receive ERT

AAV, adeno-associated viral; CRIM, cross-reactive immunological material; e2w, every two weeks; eow, every other week; ERT, enzyme replacement therapy; *GAA*, acid-α-glucosidase; HSAT, high sustained antibody titers; ITI, immune tolerance induction; NA, not available; NBS, newborn screening.

Of the 54 included articles, a total of 32 articles used CRIM testing; 66% (*n =* 21) of the studies were conducted in North America or Europe. The primary CRIM testing method used in the studies to ascertain patients’ CRIM status was Western blot analysis of skin fibroblasts (43). Presently, the label for ALG provides details on the influence of CRIM testing and ITI on patient outcomes (64, 65). However, the EU and US labels for AVA do not provide guidance regarding CRIM testing (13, 66).

#### 3.3.2 CRIM status prediction

CRIM status was predicted using detailed genetic analyses facilitated by the Pompe variant database accessible via the internet (67). GAA pathogenic variants were identified through comparison with the GenBank reference DNA sequence (Accession: NM_000152) (68). Pathogenicity assessments of missense mutations utilized the PolyPhen-2 program, and splice-site changes were evaluated using the Berkeley Drosophila Genome Project’s tool (68). CRIM status prediction was reported in 33 articles post-2016 and utilized GAA mutational analysis, thus indicating a recent trend towards the increased use of mutational analysis. This may be due to improved understanding of the role of CRIM status prediction in early treatment initiation, overall improvements in DNA sequencing, and increased cataloguing of pathogenic variants. Most of the 33 included articles (CRIM status testing: 65.6%, *n =* 22; CRIM prediction: 69.7%, *n =* 23; CRIM testing and prediction: 69.2%, *n =* 23) were from Europe and the US.

#### 3.3.3 Use of both CRIM testing and prediction

Thirteen articles post-2016 reported CRIM testing and prediction to determine the CRIM status in patients with IOPD. Of these, the majority (69%, *n =* 9) have reported studies that were conducted in the US or Europe, and a small proportion (31%, *n =* 4) in Asia and other unspecified regions (49, 69). One study reviewed patient data from treatment centers and national audits, whereas another study utilized variant analysis for rapid CRIM status determination, confirmed through western blot analysis in cases where direct testing was unavailable (31, 45).

### 3.4 CRIM status in decisions on ITI use (research question 2)

Of the 70 articles reviewed, 73% (*n =* 51) focused on decision-making and rationale or guidance regarding ITI in IOPD treatment strategies (**Figure 1B**). The key studies for research question 2 are summarized in **Table 3**.

**Table 3:** Summary of key studies for research question 2.

Author (year)	Number of patients	CRIM status	Dose	ITI regimen	Outcomes
Abbott et al. (2011) (57)	Case report	Negative	20 mg/kg eow	NA	New pattern of immunological response to ERT in a severely affected Pompe disease patient
Berrier et al. (2015) (47)	20	Negative	Cumulative dose of 20–40 mg/kg e2w	NA	Immune responses in CRIM-negative patients receiving ERT monotherapy
Desai et al. (2019) (70)	37	Positive	20–40 mg/kg eow	NA	Anti-rhGAA IgG antibody response in all CRIM-positive patients with IOPD from all previous rhGAA clinical trials, who had received ERT without immune modulation
Chien et al. (2020) (21)	28	Positive	Variable 10–40 mg/kg eow to 20–40 mg/kg/week	NA	Efficacy of rhGAA therapy
Li et al. (2021) (37)	20	Negative	Variable, 20–40 mg/kg eow to 30–40 mg/kg weekly	Rituximab, methotrexate, and IVIg	Early treatment with ERT+ITI can transform the long-term CRIM-negative IOPD phenotype
Curelaru et al. (2022) (71)	Case report	Negative	20 mg/kg eow increased within a month to 40 mg/kg/week	First course with rituximab, methotrexate, and IVIg. Second course of immunomodulation with bortezomib and maintenance rituximab and methotrexate	Outcomes with a combination of high-dose weekly ERT and an adjusted immunomodulation regimen to prevent the development of HSAT

All studies included patients with ERT-experienced status.

CRIM, cross-reactive immunological material; e2w, every 2 weeks; eow, every other week; ERT, enzyme replacement therapy; HSAT, high sustained antibody titers; IgG, immunoglobulin; IOPD, infantile-onset Pompe disease; ITI, immune tolerance induction; IVIg, intravenous immunoglobulin; NA, not available; rhGAA, recombinant human acid alpha-glucosidase.

Substantial contributions were provided by two studies, Desai et al. (70) and Li et al. (37), who examined the impact of prophylactic ITI with rituximab, methotrexate, and IVIg in CRIM-negative patients (35, 37) and a small subset of high-risk CRIM-positive patients (variants susceptible to the development of high and sustained antibody titers [HSAT] or sibling history of HSAT) (35). The studies found benefits of ITI in ERT-naïve settings in terms of both safety and effectiveness (35, 37). Some adverse effects of ITI were reported; however, the use of ITI was not discontinued, and overall, the adverse effects were transient. In a study by Desai et al. (35), out of 25 patients, five CRIM-negative patients required treatment with antibiotics and two required central line removal in two patients. Similarly, Chen et al. reported treatable infection episodes and transient symptoms like numbness and diarrhea in a couple of patients (30).

Several articles mentioned the use of ITI regimens prophylactically and in patients with CRIM-negative status. A case study of CRIM-negative patients with IOPD reported that a regimen of high-dose weekly ERT (40 mg/kg) combined with ITI may lead to favorable clinical outcomes by preventing the development of HSAT (71). Among articles that described the use of ITI in CRIM-positive patients, a specific case of sibling history leading to prenatal diagnosis of a younger sibling was noted (44). This resulted in better clinical outcomes in younger siblings due to prophylactic ITI initiation simultaneously with ERT (48). ITI was recommended in cases where a variant puts a patient at an increased risk of developing HSAT, potentially triggering an immune response to ERT treatment (72).

Fifteen articles reported non-use of ITI, primarily due to CRIM-positive status (46) or the family not consenting to initiate ITI therapy (57). CRIM-negative patients who do not receive ITI often experience rapid disease progression (47).

### 3.5 Association between ITI regimens and CRIM status (research question 3)

Among the identified articles (*n =* 69) (**Figure 1C**), 64% (*n =* 44) reported rituximab, methotrexate, and IVIg (*n =* 29, all three in combination) as the most commonly used drugs in ITI regimens for patients with IOPD. Three studies with relatively larger sample sizes reported the effectiveness of ITI therapies, including rituximab, methotrexate, and IVIg, in CRIM-negative and CRIM-positive patients with IOPD (36, 49, 73). A subset of these patients had higher doses or early treatment with ERT and ITI, which has been reported to be associated with better clinical outcomes, such as increased ventilator-free survival rates (74).

Individual reports of ITI treatment alone or in combination mostly included rituximab (*n* = 39), methotrexate (*n* = 37), and IVIg (*n* = 34). The use of bortezomib (*n* = 6), sirolimus (*n* = 3), cyclophosphamide (*n* = 3), methylprednisolone (*n* = 1), or rapamycin (*n* = 1) in an ERT-experienced setting was reported less often. The key studies for research question 3 are summarized in **Table 4**.

**Table 4:** Summary of key studies for research question 3.

Author (year)	Number of patients	Dose	ITI regimen	Outcomes
Stenger et al. (2015) (75)	2*	20 mg/kg every 2 weeks	Rituximab, methotrexate, and IVIg with bortezomib	Bortezomib-based ITI regimen can decrease antibody levels
Kazi et al. (2017) (36)	19*	20–40 mg/kg weekly or biweekly	Rituximab, methotrexate, and IVIg	Long-term safety of prophylactic ITI in the ERT-naive setting (ERT+ITI) in a large cohort
Rairikar et al. (2017) (76)	Case report*	NA	Rituximab, methotrexate, and IVIg with bortezomib	Improvement in motor abilities and reduction in antibody titers following a high-dose IVIg regimen after failing to tolerate an ITI regimen with rituximab, methotrexate, and IVIg
Broomfield et al. (2018) (58)	Case Report**	ERT dose varied from 20 mg/kg eow to 20 mg/kg/week (initial 3 months), followed by 20 mg/kg eow and 40 mg/kg/week	Rituximab, methotrexate, and IVIg	ERT treatment experience in a cohort of CRIM-negative patients
Poelman et al. (2019) (39)	3**	20 mg/kg eow to 40 mg/kg weekly	Rituximab, methotrexate, and IVIg	Survival outcomes (ventilator-free survival) in CRIM-negative patients
Yang et al. (2023) (74)	33**	Variable	Hydrocortisone	Outcomes of very early ERT with premedication hydrocortisone in patients with IOPD and compared findings with previous studies

*ERT-naïve; **ERT-experienced.

CRIM, cross-reactive immunological material; eow, every other week; ERT, enzyme replacement therapy; IOPD, infantile-onset Pompe disease; ITI, immune tolerance induction; IVIg, intravenous immunoglobulin; NA, not available.

#### 3.5.1 CRIM status and use of ITI

Of the 45 articles, a higher proportion of articles focused on CRIM-negative (58%, *n* = 26) than CRIM-positive patients (42%, *n* = 19). Most studies (81%, *n* = 21) of CRIM-negative patients, and a lower proportion (47%, *n* = 9) of CRIM-positive patients mentioned ITI. Rituximab, methotrexate, and IVIg-based ITI regimens were more commonly described in articles that focused on CRIM-negative patients (46%, *n* = 12) than CRIM-positive patients (11%, *n* = 5). Kazi et al. mentioned the use of a transient low-dose methotrexate (TLDM) protocol as an ITI treatment in CRIM-positive patients (32). Additionally, in 24 articles that included both CRIM-negative and CRIM-positive patients, the majority (75%, *n* = 18) mentioned ITI use, predominantly utilizing rituximab, methotrexate, and IVIg-based regimens.

#### 3.5.2 Clinical outcomes

Overall, studies have indicated that ITI therapies combining a short course of rituximab, methotrexate, and IVIg have been effective in mitigating antibody titer, a critical factor for treatment success, in both CRIM-negative and CRIM-positive patients (35, 38, 50, 70). IVIg continued until B-cell recovery (till CD19 count increased). After 52 weeks of treatment, a study on CRIM-negative patients treated with ERT alone with no ITI clearly showed an attenuated response to the enzyme in all outcome measures, including a significant decrease in survival, invasive ventilation-free survival, reduced improvement in cardiac response, and regression of motor milestones compared with CRIM-positive patients (27). In a Dutch study, patients with IOPD (*n* = 18, CRIM-positive [*n* = 13] and CRIM-negative [*n* = 5]) who received ERT (40 mg/kg every week, age at last follow-up 6.0 years [3.1 to 8.3 years]) plus ITI had higher ventilator-free survival (100%) than patients who received ERT monotherapy (86%, age at last follow-up 3.8 years [3.0 to 4.8 years]) (73).In total, five out of 20 articles on respiratory function reported ventilator-free survival after receiving treatment with ITI (35, 36, 61, 73, 74).

Among the studies that reported data on mobility, muscle strength, or motor function on CRIM-negative and CRIM-positive patients, improvement with ITI and ERT was reported when compared to ERT monotherapy, especially in CRIM-negative patients and CRIM-positive patients who developed HSAT (30, 50).

Evidence suggests that rituximab, methotrexate, and IVIg may be associated with positive cardiac outcomes (37). Several studies have reported a reduction in left ventricular mass index (LVMI) after receiving ERT plus ITI, despite the CRIM status in patients with IOPD (77–80). Although a difference in cardiac outcomes based on CRIM status was not noted, one study reported that a reduction in LVMI continued in patients who had received ERT plus ITI vs. ERT alone (36). Another study noted that CRIM-negative patients with IOPD experienced LVMI normalization after ITI was initiated along with ERT (75). This may be attributed to ERT being more effective in the absence of ADAs.

Biomarker data, as reported in 11 articles (21, 32, 35–37, 39, 46, 76–78, 81), highlighted the effectiveness of early intervention in the management of IOPD, particularly the role of ITI in enhancing ERT outcome by reducing the risk of immunogenic response. Evidence suggests the benefit of early treatment initiation with ERT and ITI in CRIM-negative patients in improving biomarkers, such as creatinine kinase and urinary glucose tetrasaccharide, compared to those treated later (37).

Bortezomib (34, 39, 48, 71, 75, 82), cyclophosphamide (40, 51, 83), methylprednisolone (43), and rapamycin (39) were less commonly reported in the studies included in the current SLR (*n* = 12 articles) as part of ITI regimens in ERT-experienced patients with IOPD. Despite initially good clinical effect with ERT and immunomodulation, a second course of immunomodulation with bortezomib followed by a maintenance regimen with rituximab and methotrexate was required in certain patients to decrease the antibody titers. This establishes the successful use of a bortezomib-based regimen (34, 71, 75).

### 3.6 Key recommendations from published guidelines

This section summarizes the key recommendations from previously published expert panels based on the systematic evidence by Pascual-Pascual et al. (84) (Spanish expert panel), Gragnaniello et al. (85) (Italian expert panel), and Al-Hassnan et al. (5) (expert panel from the Gulf region published in October 2022 [after the search period for the SLR; it has been summarized because of its relevance]).

The key recommendations are mentioned below:

- CRIM status should be determined before initiating ERT since CRIM-negative patients have a very high risk of developing an immune response against ERT, potentially leading to treatment failure (5, 84, 85).
- CRIM status can be determined by analysis of the genetic variants (86) in >90% of the patients with IOPD with a turnaround time of less than a week (5, 84, 85).
- Western blot analysis of proteins from blood is a rapid method for determining CRIM status (within 48–72 hours of sample collection) (63). When combined with genetic prediction, western blotting can lead to well-informed treatment decisions, often within a week. This method is appropriate when there is doubt about the CRIM status based on genetic analysis. Western blot analysis of GAA in skin fibroblasts has been used in the past for CRIM status determination; however, it can take longer to obtain results (85).
- Initiating ITI therapy concurrent or prior to the first dose of ERT can improve the prognosis of patients with IOPD with either CRIM-negative or CRIM-positive status (85).
- ERT can significantly improve clinical outcomes when treatment is initiated promptly after diagnosis (84). Early initiation of ERT in IOPD has been associated with improved survival and motor functions in a cohort of patients with an average age at ERT initiation of 9.75 ± 3.17 days (74). Prophylactic ITI regimens can be used to preempt immune response in CRIM-negative and CRIM-positive patients in an ERT-naïve setting (85). Nearly 32% of the CRIM-positive patients develop HSAT. It is difficult to predict which CRIM-positive patients have a high risk; hence, a prophylactic ITI regimen is preferred based on clinician insights (33, 70).
- ITI regimen in an ERT naïve setting in IOPD includes a 5-week regimen of rituximab and methotrexate plus monthly IVIg (until B cell recovery) in CRIM-negative and CRIM-positive patients (85). A TLDM can also be used in patients with CRIM-positive disease (32).
- The clinical condition of the patient, or the presence of other risk factors, may prompt consideration of the risks and benefits of ITI in an individual patient. In certain very severe cases, it is recommended to start ITI before CRIM status is known.
- Recommendations from an expert panel from the Gulf region suggest that combining ITI with ERT could provide substantial clinical benefit, particularly in CRIM-negative patients (who make up a high proportion of patients with IOPD in the region), but that evidence is limited (5).
- CRIM status can often be predicted from GAA variants or confirmed by Western blot when the association between variants and CRIM status is unclear. However, limited test availability and a lack of globally standardized protocols for Western blot analysis for CRIM status and criteria can affect consistent CRIM-status classification. Despite this, assessment of CRIM status should not delay the initiation of treatment when IOPD is confirmed or highly suspected (35).
- Implementing ITI concurrent with ERT initiation according to the protocol described by Banugaria et al. (31) for all patients with IOPD, particularly CRIM-negative patients, along with high-dose ERT, significantly improves outcomes and reduces mortality and morbidity.
- Regular monitoring and quantification of anti-rhGAA antibodies at baseline and throughout the treatment are required to assess the need to repeat the ITI regimen (34). The threshold for HSAT is different in patients treated with different doses of ERT (29, 87).

### 3.7 Key findings and expert opinion

The findings from the current SLR agree with the available global recommendations, indicating the increased use of CRIM status prediction, along with formal testing, to identify patients at risk of developing high antibody titers. ITI regimens are beneficial and have been extensively used in CRIM-negative patients. Regimens, notably those including rituximab along with methotrexate and IVIg, have led to clinical improvements, such as reduced antibody titers and mortality, improved mobility, and respiratory and cardiac functions, in addition to positive changes in biomarkers. Increased NBS, followed by swift CRIM status determination, enables early initiation of ITI in conjunction with ERT, especially in CRIM-negative patients. This leads to better clinical outcomes and better overall survival for most patients.

#### 3.7.1 Expert suggestions

- ERT initiation: ERT should be initiated as early as possible. Higher doses of ERT have been shown to be beneficial.
- Timely treatment: Assessing CRIM status should not delay the initiation of treatment when IOPD is confirmed or suspected (71).
- Using blood for CRIM status determination: Determination of CRIM status in fibroblasts is not time-efficient due to the interval of weeks needed to culture fibroblasts; this method is not suitable in clinical practice. However, in some situations, it is good to do this in the setting of novel variants and inconclusive results on blood CRIM testing. This should, however, not result in a delay in treatment, and ITI should be initiated. The prediction of CRIM status based on the patient’s *GAA* mutation or testing in blood should be done in accredited and licensed clinical laboratories with validated methodologies. Interpretation of results should be very careful in reference to experts.
- ITI with unclear CRIM status: If a patient’s CRIM status cannot be predicted using mutational analysis, or if there is a delay in getting variant reports, the use of ITI with unclear CRIM status is suggested. However, this should be handled with caution by expert centers.
- ITI Protocol implementation: ITI was implemented according to the protocol described by Banugaria et al. (31) for all patients with IOPD, particularly for CRIM-negative patients. High-dose ERT, in conjunction with ITI, significantly improved outcomes and reduced mortality and morbidity (20). ITI with transient low-dose methotrexate can be considered in CRIM-positive patients with IOPD (35).
- Safety of ITI regimens: ITI regimens have been found to be well-tolerated based on published evidence (9, 29) and clinical experience.
- Regular Monitoring: Regularly monitor and quantify anti-rhGAA antibodies at baseline and throughout treatment. Repetition of ITI should be based on these assessments (34).
- HSAT risk in CRIM-positive patients: Predicting the risk of developing HSAT in most CRIM-positive patients is difficult (88). However, if ITI is administered simultaneously with the first ERT infusion, patients may develop lower levels of ADAs.
- Management of HSAT: In cases of HSAT, management with longer-term immunomodulatory treatment, including bortezomib, methotrexate, and rituximab, or other B-cell and plasma cell agents, is needed.

## 4 Discussion

This SLR summarizes the published evidence and provides guidance on CRIM status, its impact in a clinical setting, testing and prediction, and various treatment regimens used in real-world practice to improve the outcomes of ERT-naïve patients with IOPD. The publications identified (from January 1, 2003, to August 4, 2022) provide a comprehensive overview of the evolution of CRIM status determination and ITI regimens used for patients with IOPD.

While examining the impact of CRIM status on patients, the recent increase in the understanding of the importance of NBS in the early detection of IOPD is noteworthy. Prenatal screening and NBS can lead to early identification of patients before significant clinical manifestations develop, facilitating early treatment initiation (21, 59). Initial studies focusing on NBS were primarily concentrated in Taiwan, the US, and Italy (37, 46, 59, 69, 77, 89). There has been evidence indicating that antibodies develop in early-treated patients within the first month of life, irrespective of CRIM status (90). Furthermore, long-term waiting for a CRIM status should be avoided. It is recommended to initiate ITI in severe cases.

ERT, as a primary treatment modality, has been the focus of IOPD (71, 91). However, the effectiveness of ERT depends on many factors, including the patient’s CRIM status (50), early age at ERT initiation (92) and dose administered (71). Patients may develop ADAs to ERT, which affects the overall efficacy of ERT in managing disease manifestations (33). Established ADAs are more commonly noted in patients with CRIM-negative status (24). Evidence suggests the development of ADAs in infants, leading to neutralized outcomes of ERT, thus necessitating the use of immunomodulation therapy (40, 93). Up to one-third CRIM-positive patients can also develop antibodies; hence, there is a need to recognize them either by using a prophylactic approach or by close monitoring (88). There is a need for regular monitoring of antibody titers as patients with high and sustained antibody titers (HSAT) can lose the therapeutic benefits of enzyme replacement therapy (ERT) (30, 34). Desai et al. reported the median time of the development of high HSAT since ERT to be 10 weeks (range, 4–24 weeks), with an upward trend within the first 24 weeks (34). Banugaria et al. have developed an algorithm to rapidly diagnose CRIM-negative patients with IOPD and determine and initiation of an ITI regimen along with ERT at the earliest possible time point (5, 31).

Additionally, it is important to recognize that in rare instances, patients showing ERT tolerance may have a break in tolerance and develop HSAT. In an LOPD patient who was initiated on ERT at age 20 months, there was a breach in tolerance after 11 years on ERT, minimize delays between CRIM status (94). The CRIM status determination has evolved over time. Traditionally, western blot analysis of skin in skin fibroblasts and GAA sequencing or genetic testing was undertaken for the CRIM status determination and has been pivotal in adapting treatment strategies (63). However, this process is time-consuming and delays the initiation of therapy. A blood-based CRIM assay is now performed, which is less time-consuming (49, 61, 62). Genetic testing reveals specific GAA variants, with guiding predictions of CRIM status in ~92% of patients (26); a combination of blood CRIM and mutation analysis has also been reported (71). This has prevented delays as blood testing CRIM results can be performed in 48 to 72 hours. Moreover, this offers rapid treatment decisions and augments clinical outcomes in patients with IOPD. In case of variants with an unknown effect on CRIM status, testing of CRIM status should be performed in an expert center. Currently, genetic testing is the preferred method, as it identifies specific variants and guides more precise treatment decisions. Nonetheless, detailed genotypic information is critical for optimizing long-term management, including the application of ITI in CRIM-negative cases to increase ERT effectiveness (36).

CRIM-negative patients require ITI regimens in the prophylactic setting (i.e., at the time of ERT initiation) to prevent the development of HSAT antibody formation against ERT (36, 39), whereas CRIM-positive patients with IOPD largely exhibit a more favorable response to ERT owing to lower immunogenicity, among other factors, generally resulting in less severe immune responses (21). However, a subset of CRIM-positive patients may elicit a stronger immune response similar to CRIM-negative patients (28, 70). In the real world, administering a TLDM protocol to high-risk CRIM-positive patients with IOPD and, in some instances, CRIM-negative patients have been used. TLDM is particularly used where rituximab is not readily available and is also based on an acceptable safety profile, although requiring long-term follow-up data (32). Notedly, ITI regimens using rituximab, methotrexate, and/or IVIg were well-tolerated in many patients with IOPD (35).

Early initiation of ERT for IOPD has shown promising results in improving the outcomes of therapy, especially cardiomyopathy, motor status, and respiratory status (84, 85, 95). The effectiveness of ERT and ITI in treating IOPD has been extensively documented as being safe and tolerable. Several reports have documented the successful use of ITI protocols to mitigate the immune response against ERT, addressing the challenges of immunogenicity, particularly in CRIM-negative patients (35, 75, 96). Evidence suggests that ITI regimens using rituximab, methotrexate, and IVIg in ERT-naïve patients with IOPD are generally well-tolerated (36, 47). In a study by Desai et al. (35), five CRIM-negative patients required treatment with antibiotics and two required central line removal. Similarly, Chen et al. (30) reported treatable infection episodes and transient symptoms like numbness and diarrhea in a couple of patients. In all these cases the ITI course was completed. A recent case study found that early initiation of bortezomib-based regimens was successful in patients that broke tolerance despite prophylactic ITI (34).

While the use of ITI in CRIM-positive IOPD patients has been less consistent compared to CRIM-negative patients, likely due to varying clinical practices and perceived lower risk, the potential for improved outcomes with prophylactic ITI supports its consideration as an important strategy even in CRIM-positive cases. Further research is warranted to better define its role (35, 37). In addition to early treatment and antibody management, the use of higher doses of ERT has been identified as a critical factor contributing to the improved treatment outcomes (37). A study published in 2024 demonstrated that bortezomib, along with rituximab, methotrexate, and IVIg, was successful in reducing high sustained ADA titers (30). It is important to understand how ITI agents are used for treatment; for example, bortezomib is used in the ERT-experienced patients.

The inclusion of a comparison between patients who received ITI at the initiation of ERT and those who received ITI after high ADA levels have developed is critical to understand the impact of ITI timing on the efficacy and safety of ERT in naive patients. This evidence, while present, is not currently obtained from a systematic, prospective data collection and requires retrospective analysis and expert consensus to fill this gap. Future research should be directed toward specifically designed studies to address this important clinical question. This approach will help provide a comprehensive understanding of the optimal timing of ITI in the context of ERT, ultimately leading to improved patient outcomes and more informed clinical practice. For individualized treatment regimens, factors such as the age at diagnosis, family history, HLA-binding predictions, GAA genotype, and CRIM status offer the prospect of improved patient outcomes (24).

A review by Desai et al. noted that available publications, including immunomodulation, reported the use of various clinical endpoints, making it difficult to compare the effectiveness of various immunomodulation strategies (33). Similar barriers to comparison were noted in the current review. The included articles reported diverse patient data in terms of age, sex, clinical presentations, dose level, and frequency of treatment, indicating that comparisons between patient groups, i.e., ERT-naïve and ERT-experienced, were not feasible. The review only provided a qualitative synthesis of the findings, without statistical analyses. The statistical analysis could not be undertaken because of the variability in the included publications, which was outside the scope of the present review. Most of the studies included in this review had small sample sizes, which is often a challenge for rare diseases.

## 5 Conclusions

Most studies on CRIM testing and prediction have been conducted initially in the US and subsequently in Europe. Recently, there has been a significant shift from direct CRIM testing using western blotting to CRIM status prediction based on genetic mutational analysis. The use of ITI, particularly in CRIM-negative patients, was noted to minimize the impact of ADAs on the treatment response. Overall, the benefits of early diagnosis and intervention are significant, particularly in patients with a family history of Pompe disease. Early treatment initiation can improve patient outcomes, underscoring the importance of genetic counseling and family history assessments in managing Pompe disease.

Large-scale studies with standardized endpoints are warranted to generate unified guidance regarding ITI regimens in CRIM-positive and CRIM-negative patients. Uniform guidance can then be tailored to meet the region-specific requirements. Additionally, there is a need for guidance supported by real-world evidence regarding treatment strategies based on the CRIM status to assist clinicians in managing patients with IOPD.

## Data Availability

The datasets presented in this article are not readily available because the datasets used and/or analyzed during the current study are available from the corresponding author upon reasonable request. Requests to access the datasets should be directed to PK, priya.kishnani@duke.edu.
